# Protocol for assessing affective responses in caregiver-infant dyads during the still-face procedure using infrared thermal imaging

**DOI:** 10.1016/j.xpro.2025.104281

**Published:** 2025-12-19

**Authors:** Nicolás Hinrichs, Sofia Attolini, Mirko Morgese Zangrandi, Sarah Nazzari, Livio Provenzi

**Affiliations:** 1 Research Group Cognition and Plasticity, Max Planck Institute for Human Cognitive and Brain Sciences, 04103 Leipzig, Germany; 2 Embodied Cognitive Science Unit, Okinawa Institute of Science and Technology Graduate University, 904-0495 Okinawa, Japan; 3 Department of Psychiatry and Psychotherapy - Campus Benjamin Franklin, Charité – Universitätsmedizin Berlin, 12203 Berlin, Germany; 4 Department of Psychology, University of Milan-Bicocca, 20126 Milan, Italy; 5 Department of Brain and Behavioral Sciences, University of Pavia, 27100 Pavia, Italy; 6 Developmental Psychobiology Lab, IRCCS Mondino Foundation, 27100 Pavia, Italy

**Keywords:** Developmental biology, Health Sciences, Neuroscience, Behavior

## Abstract

Here, we present a protocol for assessing thermal affective responses in caregiver-infant dyads during the still-face procedure (SFP) using infrared thermal imaging (ITI). We describe steps for preliminary experiment design and setup and data collection. We then detail a pipeline to preprocess and analyze dual ITI data and discuss relevant technical issues. We also provide possible future adaptations of this protocol to study natural interactive exchanges in typical and atypical pediatric populations.

For complete details on the use and execution of this protocol, please refer to Nazzari et al.^1^

## Highlights

• Steps to monitor caregiver-infant emotion with infrared thermal imaging
• Instructions for dual thermal camera setup and synchronized video recording
• Guidance to measure autonomic and behavioral regulation in the still-face task
• Procedures for thermal preprocessing and facial ROI extraction for time series

## Abstract (editor highlights)

Publisher’s note: Undertaking any experimental protocol requires adherence to local institutional guidelines for laboratory safety and ethics.

## Graphical abstract

**Figure:**
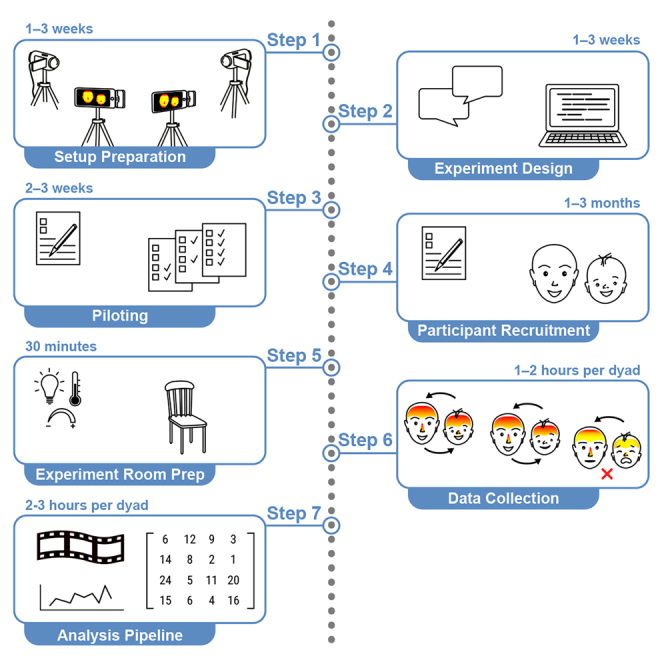

## Before you begin

In the past two decades, the application of physiological measurements to study social interaction research has expanded significantly, offering more profound insights into the underlying mechanisms of early development.^2^ Historically, such investigations were constrained to studying the behavior and physiology of single participants, thus neglecting the inherently dyadic or group-based nature of social interactions. Infrared Thermal Imaging (ITI) has emerged as a valuable non-invasive method, particularly well-suited for capturing physiological activity during naturalistic interactions without needing physical contact.^1^ By measuring subtle modulations in skin temperature, ITI provides a direct window into autonomic activity and allows differentiation between psychophysiological arousal and baseline states.^3^ In particular, ITI studies have focused on thermal responses on facial regions of interest (ROIs), such as the tip of the nose and the forehead,^4^ to explore physiological reactions to affective stimuli. In recent years, a few studies have explored thermal responses during caregiver-infant interactions,^2^^,^^5^ highlighting their potential in investigating affect during early interactions.

The Still-Face Procedure (SFP)^6^ is a well-established three-phase paradigm (i.e., Play, Still-Face, Reunion) that allows researchers to explore naturalistic caregiver–infant interactions. It provides a controlled framework for assessing emotional regulation, stress responses, and co-regulatory dynamics in early development^7^ and has been employed to investigate psychophysiological processes during caregiver–infant interactions.^8^

When combining ITI with the SFP, three methodological considerations are essential. First, technical constraints must be addressed: ITI registers autonomic shifts with a temporal latency that is not yet fully established, but appear to be longer compared to traditional indices of autonomic activity such as skin conductance.^4^^,^^5^ Furthermore, it is sensitive to motion artifacts, limited camera sensitivity, and fluctuations in ambient temperature. To ensure data reliability, recordings should be conducted in a thermally stable environment, with the ambient temperature preset and maintained at a constant value within a 18–24 °C range. Motion artifacts can be minimized through frame-rejection criteria or the use of motion-compensation algorithms. The selection of a thermal camera model should be carefully considered to ensure sufficient sensitivity and resolution for detecting subtle autonomic changes. Second, the evidence base is steadily growing, with studies^9^^,^^10^ pairing ITI with fine-grained behavioural coding showing that facial temperature reliably mirrors affective downturns during the Still-Face episode, distinguishes caregiver-versus-stranger exchanges, and (when synchronised across dyad members) informs caregiver–infant co-regulation dynamics. Third, the added value of integrating ITI into the SFP lies in its ability to provide a contact-free physiological complement to behavioural observation, capturing covert autonomic shifts precisely when overt signs such as gaze, affect, and self-comforting unfold.

This protocol employs high-resolution, dual-camera ITI, integrated with synchronised video recordings for behavioural coding during the SFP. Detailed guidance on equipment setup, participant preparation, and data collection ensures consistency and scalability. The protocol can be adapted to variations such as technoference or paperference SFP,^1^ or other structured tasks (e.g., guided play, problem-solving) that allow comparison of technology-specific versus generic caregiver disengagement, or manipulation of leadership roles in the interaction.

### Innovation

This protocol advances the study of caregiver–infant interaction by integrating high-resolution, dual-camera ITI with the SFP in a synchronized, multi-modal recording framework. While both ITI and SFP have been used independently, and Nazzari et al.^1^ demonstrated their combined feasibility in a modified SFP, the present protocol extends that work by formalizing step-by-step procedures for environmental control, camera configuration, and participant handling to ensure reproducibility across laboratories. The setup is optimized for early developmental populations, with precise guidelines for ambient temperature stabilization, thermal radiation management, and seating arrangements that maintain consistent face visibility. A key innovation is using synchronized, LWIR cameras (one per participant) combined with parallel RGB video for behavioural coding, ensuring complete face coverage for both dyad members and enabling cross-participant analyses of physiological synchrony. Experimental flexibility is expanded through validated variations such as technology-based (technoference) and non-digital (paperference) distractions, allowing investigation of caregiver engagement across modern and traditional contexts. On the analytical side, the protocol introduces an open Python pipeline for frame-by-frame temperature extraction from radiometric images, featuring interactive ROI selection, motion artefact mitigation, and consistent temperature scaling; this customizable approach facilitates reproducible analysis across ITI systems and research groups. By merging a validated social stress paradigm with rigorous environmental controls, dual-camera ITI acquisition, and transparent, open-source analysis tools, this protocol delivers a scalable, reproducible framework for investigating the dynamics of autonomic–behavioural coupling in both typical and at-risk caregiver–infant dyads.

### Institutional permissions

This protocol is based on the study by Nazzari et al.^1^ involving human participants, which investigated how digital and non-digital caregiver distractions influence mother–infant behaviour and facial temperature in a modified SFP. The study was conducted in accordance with the Declaration of Helsinki and approved by the Ethics Committee of the local institution. All procedures, including ITI and behavioral video recordings, are designed to be non-invasive and prioritize participant well-being. Before carrying out a study following this protocol, its use must be: 1) approved by an institutional review board, specifying the use of contactless measures and their relevance to studying physiological and behavioral responses in caregiver-infant dyads, 2) conducted in compliance with GDPR or equivalent regulations by assigning anonymized identifiers, removing personally identifiable information, and restricting access to raw data to authorized personnel, and 3) managed with clear data retention policies, allowing participants to request data deletion and ensuring that de-identified datasets (e.g., via Open Science Framework) are shared only with proper consent for research transparency.

## Key resources table

**Table:** 

REAGENT or RESOURCE	SOURCE	IDENTIFIER
**Deposited data**		

Exemplary data and scripts	This paper	https://osf.io/8pfx9/
Data and code	This paper	https://osf.io/8pfx9/

**Experimental models: Organisms/strains**		

38 3- to 5-month-old infants (mean age = 3.97 months, SD = 0.79, mostly white) and their caregiver	Nazzari et al.^1^	https://doi.org/10.1016/j.biopsycho.2025.109027

**Software and algorithms**		

*Visual Studio Code* (*version 1.82*)	Microsoft Corporation (2023). Visual Studio Code (version 1.82).	https://code.visualstudio.com/
*Python* (*version 3.11.4*)	Python Software Foundation (2023). *Python* (*version 3.11*).	https://www.python.org/
*Python package - opencv* (*version 4.8.0.76*)	OpenCV Team (2023). *OpenCV* (*version 4.8.9*)	https://opencv.org/
*Python package - NumPy (version 1.25.2*)	NumPy Developers (2023). *NumPy* (*version 1.25.2*)	https://numpy.org/
*Python package - matplotlib-pyplot* (*version 3.7.5*)	Matplotlib Development Team (2023). *Matplotlib* (*version 3.7.5*)	https://matplotlib.org/
*Python package - pandas* (*version 2.1.0*)	Pandas Development Team. (2023). *pandas* (*version 2.1.0*)	https://pandas.pydata.org/
*Python package - TkInter* (*version 8.6*)	Python Software Foundation (2023). *Tkinter: Standard GUI toolkit for Python*. In *Python Documentation* (version 3.11)	https://docs.python.org/3/library/tkinter.html
*Microsoft Excel 2021* (*version 17.0*)	Microsoft Corporation (2021). *Excel* (*Microsoft 365 Subscription*). [Computer Software]. Microsoft Corporation.	https://www.microsoft.com/en-us/microsoft-365/excel#:∼:text=Microsoft%20Excel%20with%20a%20Microsoft,Excel%202007%2C%20and%20Excel%202003.
*Jamovi Desktop* (*version 2.4*)	The jamovi project (2023). *jamovi* (version 2.4) [Computer Software]	https://jamovi.org

**Other**		

Samsung Gear 360 Action Cam	Samsung	https://www.samsung.com/it/support/model/SM-C200NZWAITV/
Two FLIR ONE PRO thermal cameras with streaming capacity	TELEDYNE FLIR	https://www.flir.it/products/flir-one-pro/?model=435-0007-03&vertical=condition+monitoring&segment=solutions
Two Smartphones	Android	-
Two tripods for smartphones	MANFROTTO	https://amzn.eu/d/3jq0Xgh
Two tripods with horizontal arm for cameras	NEEWER	https://amzn.eu/d/ejesFLc
Removable Black curtains		https://amzn.eu/d/e8Mbobl

## Step-by-step method details

### Experiment design

**Timing: 1–3 weeks**

This section outlines the conceptual and methodological decision required before data collection begins. It covers defining research objectives, participant eligibility, experimental conditions, and statistical considerations to ensure the study is robust and reproducible.

1. Define your research objectives and hypotheses.
  a. Clarify your main goal. Decide whether to:
    i. Characterise typical affective and physiological responses in healthy dyads.
    ii. Compare groups (e.g., clinical vs. non-clinical).
    iii. Test an intervention’s effect on stress regulation.
  b. Formulate specific, testable hypotheses.
    Define expected changes in thermal and behavioral indices across SFP phases and/or between groups.
    ***Note:*** Aligning your experimental questions with explicit hypotheses guides inclusion criteria, sample size, and analytical strategy. Example hypotheses include decreased infant nasal tip temperature during the Still-Face vs. Reunion and associations between caregiver thermal responses and infant behavioral regulation.
2. Determine in- and exclusion criteria for participation in the study.
  a. Define infant age range.
    Typically use ∼3–9 months, when infants reliably engage in social interaction but are not yet mobile enough to disrupt the setup.
  b. Define caregiver language requirements.
    Specify the language of interest of the adult participants.
  c. Exclusion criteria for ITI studies include:
    i. Exclude infants or caregivers with impairments of cutaneous thermoregulation (e.g., peripheral neuropathy, micro- or macroangiopathies, connective tissue diseases, psychophysiological disorders).
    ii. Add or relax criteria according to your research goals (e.g., include specific clinical diagnoses, exclude medications affecting vasomotor tone).
      ***Note:*** Ensure that the caregiver are in good health and that the infant is healthy and full-term.
3. Define the structure of the Still-Face Procedure (SFP).
  Adopt the classic 3-phase SFP structure (Play → Still-Face → Reunion).
  a. Play (2 min).
    Instruct the caregiver to interact naturally (talking, smiling, gesturing).
  b. Still-Face (2 min).
    Instruct the caregiver to maintain a neutral facial expression and avoid talking, smiling, or touching.
  c. Reunion (2 min).
    Instruct the caregiver to resume normal interaction and soothe the infant as needed.
4. Set environmental and timing controls.
  a. Control room temperature and lighting.
    i. Maintain room temperature between 18° and 24°C by means of A/C, with minimal drafts and stable lighting (consult Troubleshooting problem 3 if ambient temperature fluctuates).
    ii. Avoid direct sunlight or heat sources (lamps, radiators) that may skew thermal recordings.
  b. Standardize time of day.
    Schedule sessions at similar times (e.g., midmorning) to reduce circadian variations in stress and skin temperature.
  c. Provide pre-session acclimation.
    Invite caregiver–infant dyads to arrive 10–15 min early so both can adjust to the ambient temperature (18°C–24°C) before recording.
5. Plan randomization or additional experimental conditions. If applicable, include additional tasks:
  a. Counterbalance tasks or SFP order.
    Randomize the order of additional tasks (e.g., baseline toy play, reading) relative to the SFP.
  b. Manipulate background conditions if needed.
    Add background noise (music, white noise) only if you explicitly test its effect on thermal or behavioral responses.
  c. Implement between-subject designs where relevant.
    Use validated screening instruments (e.g., maternal depression scales) to allocate participants to clinical vs. control groups.
6. Decide on sample size and perform a power analysis.
  a. Estimate effect sizes: Use prior ITI or pilot data on infant and caregiver thermal changes (and/or behavioural outcomes) to inform sample size calculations.
  b. Plan for attrition: Recruit more dyads than your target analyzable sample.
    ***Note:*** Infant studies often face higher dropout or data loss (e.g., fussiness, motion artefacts, failure to complete the SFP). Recruit approximately 20%–30% more participants than the desired final sample.
  c. Use appropriate power analysis tools: Use dedicated software (e.g., SPSS modules, G∗Power, jamovi) or consult a statistician to ensure sufficient power for within-subject effects (e.g., phase contrasts) and between-group comparisons.

### Setup preparation

**Timing: 1–3 weeks**

This section describes how to prepare the physical environment, seating arrangement, and equipment for the Still-Face Procedure with thermal imaging. The goal is to ensure optimal thermal measurement conditions and consistency across sessions.

7. Define the **location and seating arrangement** for the Still-Face Procedure (SFP):
  a. Assign research staff.
    i. Assign at least one behavioral scientist trained in administering the SFP and infant ethics.
    ii. Assign at least one technician experienced with ITI camera positioning, setup, and acquisition procedures.
  b. Select and stabilize the room.
    i. Select a quiet, private room with minimal external distractions and a stable controlled temperature (ideally 18–24°C).
    ii. Avoid placing participants near active heating/cooling vents, radiators, cold windows, or uninsulated walls.
      ***Note:*** If full temperature stability cannot be guaranteed, continuously monitor room temperature using an independent calibrated thermometer. Log any fluctuations and model them as covariates during analysis if needed.
  c. Manage thermal radiation sources.
    i. Block direct sunlight using thick curtains or blinds.
    ii. Switch off or dim heat-emitting lights (e.g., incandescent bulbs) when possible.
    iii. Ensure no active heating ducts, hot water pipes, or personal heaters are within the camera’s field of view or influencing participant/background temperatures.
  d. Define seating setup.
    i. Place two seats facing each other: one for the caregiver and one for the infant.
    ii. Select an age-appropriate infant seat (e.g., baby bouncer, high chair, stroller, car seat) that keeps the infant comfortably upright with a fully visible face.
    iii. Provide a stable chair for the caregiver directly facing the infant.
    iv. Mark chair positions on the floor with tape to maintain consistent angles and distances across dyads.
    v. Position seats so the caregiver can comfortably reach and soothe the infant within protocol constraints, typically 50–70 cm apart.
  e. Standardize seating across sessions.
    Mark each seat’s position permanently (e.g., tape) to reproduce the same setup for all participants.
    ***Note:*** Consistent lighting, seating distance, and angles are critical for comparable thermal measurements and video recordings across dyads.
8. Set up a thermally stable and non-reflecting background, if needed:
  a. Hang a non-reflective background material (e.g., a matte fabric in a neutral shade like gray or black).
  b. Maintain a sufficient distance between participants and walls or backdrops (≥1 m) to reduce the influence of reflected thermal radiation or localized hot/cold spots on surfaces not within the immediate background.
  c. Ensure caregiver comfort and infant visibility.
    i. Provide enough space for caregiver arm movements (except during Still-Face).
    ii. Instruct the caregiver to keep the infant’s face as unobstructed as possible.

***Note:*** A small table or tray for tissues or soothing items can be placed nearby, provided it does not obstruct the thermal camera’s view.

9. Configure the ITI setup (see Figure 1).
  ***Note:*** The use of two thermal cameras (one per participant) is highly recommended, one pointing at the caregiver and the one pointing at the infant. The system used in the associated study^1^ is a long wavelength infrared (LWIR) camera, which detects emitted radiation in the 8–14 μm band.
  a. Position the ITI cameras.
    i. Mount each thermal camera on a stable tripod to obtain a clear, frontal view of each face.
    ii. Set the measurement distance according to the camera’s specifications.
    iii. Test and confirm the optimal distance for your camera and ROI size through trial recordings.
    iv. Adjust tripod height so the full face is visible and the camera is approximately perpendicular to the face (normal incidence) to avoid angular distortion.
    v. Aim for the face to occupy ∼40% of the frame (acceptable range 30–50%). Continue recording if the infant turns or covers the face, but flag these frames for exclusion and repeat the episode only if more than one-third of frames are unusable.
    vi. Mark tripod locations on the floor with tape to replicate camera placement across sessions, adjusting height at each session based on participant height.
      ***Note:*** For low-resolution (e.g., 160×120 pixels) fixed-focus cameras, an approximate distance of ∼1 m often ensures sufficient pixel coverage (e.g., ≥10×10 pixels per smallest ROI) and focus. Aim for the face (chin to forehead, ear to ear) to occupy ∼30%–50% of the frame.
  b. Configure ITI camera settings.
    i. Set emissivity to 0.98 (typical for human skin).
    ii. Predefine a fixed temperature range (e.g., 17–42°C) that covers expected physiological variation.
    iii. Enable video or image streaming mode if available.
    iv. Disable dynamic display controls such as Automatic Gain Control (AGC), Auto Exposure (AE), and Auto White Balance (AWB), if possible.
      ***Note:*** Disabling these features prevents frame-by-frame rescaling based on the hottest/coldest objects and supports a stable pixel–temperature mapping. In many LWIR systems, these settings affect only the preview, but keeping them fixed also stabilizes monitoring during acquisition.
    v. Plan to assign the thermal color palette in post-processing based on measured temperature values rather than relying on the live preview.
      ***Note:*** Entry-level ITI cameras are attractive for their portability and cost but typically offer lower precision than high-end systems. As a minimum, aim for: sensor resolution ≥160×120 pixels, NETD ≤70 mK, sampling rate ≥8–9 Hz (≥15 Hz preferable), temperature range ∼17°C–42°C, and field of view ≥50°×43°. Use identical camera models (same specifications) for caregiver and infant to ensure comparability. Many uncooled LWIR cameras perform automatic Non-Uniformity Correction (NUC) at intervals; this can slightly alter preview and exported videos.
  c. Finalize ITI setup.
    i. Switch on the ITI cameras and allow them to complete their internal self-correction routine according to manufacturer guidelines.
    ii. Remove reflective surfaces (metal, glass) from the immediate field of view.
    iii. Perform a 30 s test recording with participants in position.
    iv. Verify full face visibility for both infant and caregiver; adjust height/angle if needed.
    v. Ensure sharp focus using autofocus or manual focus.
    vi. Inspect faces for spurious hot/cold spots suggesting unwanted reflection.
      **CRITICAL:** Switch on each ITI camera at least 10 min before formal data collection to allow thermal stabilization. Simultaneously, allow a 10–15 min acclimation period for participants in the room (18°C–24°C) to minimize transient skin temperature artefacts from recent environmental changes and to ensure that the infant is settled before the SFP begins. Extend this period if the infant becomes distressed and allow caregivers to soothe as needed.

**Figure 1:**
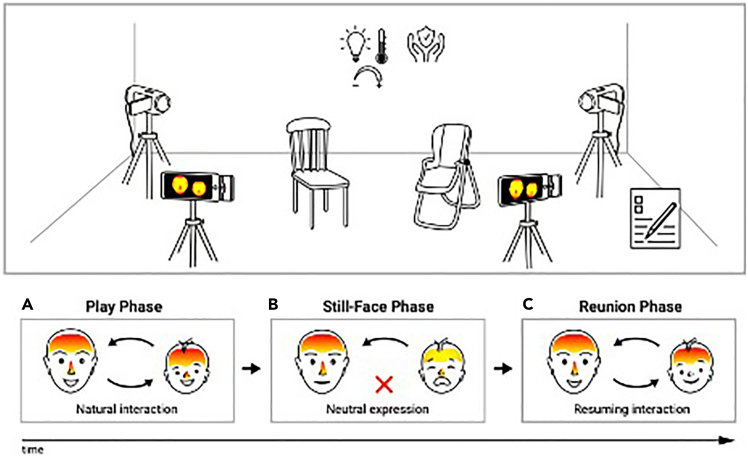
Experiment design Standard three-phase Still-Face Procedure (Play, Still-Face, Reunion), each 2 min, recorded with thermal and video cameras in a controlled room (between 18°C and 24°C). Dyads are seated face-to-face with fixed positions; a 10–15 min acclimation precedes the session.
10. Configure behavioral video and audio recording.
  a. Set up at least one standard video camera for synchronous behavioral recording.
    i. For minimal setups, place a single wide-angle or 360° camera capturing caregiver and infant together.
    ii. For more detailed analyses, use two frontal cameras (one per participant).
    iii. Confirm adequate resolution (≥1080p) and secure mounting on tripods.
    iv. If using audio, position built-in or external microphones near participants without obstructing the infant’s face in the thermal view.
      ***Note:*** Concurrent video (and audio) recordings are highly recommended for fine-grained coding of caregiver-infant interaction and for synchronizing behavioral events with thermal data.
  b. Synchronize thermal and behavioral feeds (optional but recommended).
    i. Introduce a clear synchronization cue visible in all recordings at the start (e.g., hand clap, spoken “start”).
    ii. Verify that timestamps or cues allow precise alignment during analysis.
      ***Note:*** When capturing audio, keep volumes comfortable and test microphone gain to avoid clipping and loss of quiet infant vocalizations.

#### Researcher training

**Timing: 1–2 weeks**

This section details the skills and knowledge required by research staff to execute the Still-Face Procedure with infrared thermal imaging, including technical camera operation and sensitive interaction with infants.

11. Familiarize the team with the technical aspects of ITI thermal imaging.
  a. Review proper camera calibration, placement, and temperature range settings to minimize procedural errors.
  b. Practice evaluating test recordings to identify and troubleshoot common artifacts (e.g., reflections, poor angle, motion issues).
12. Train research staff in handling infants and conducting the Still-Face Procedure.
  a. Emphasize a sensitive, infant-centered approach that balances the protocol’s aims with participants’ comfort.
  b. Provide clear guidelines on how to smoothly transition between SFP phases (Play, Still-Face, Reunion) and when to halt or modify the procedure if infants show excessive distress.

### Piloting

**Timing: 2–3 weeks**

This section explains how to conduct small-scale pilot sessions to test equipment, procedures, and participant instructions, allowing for refinement before the main study.

13. Define the goals of the pilot experiment.
  a. Specify whether the pilot will:
    i. Test equipment functioning (thermal + video setup).
    ii. Test clarity of caregiver instructions.
    iii. Evaluate preliminary thermal and/or behavioral data.
      ***Note:*** If you only need to verify equipment and instructions, 2–3 dyads are typically sufficient. For preliminary data inspection or refinement of behavioral coding, consider 5–10 dyads.
  b. Test the equipment.
    i. Verify that the thermal imaging setup (camera calibration, angle, temperature range) functions properly.
    ii. Check video and audio recording quality to ensure both caregiver and infant faces are adequately captured (see Troubleshooting, problem 1).
    iii. Confirm that any recording software (if used to monitor the thermal camera live) is working consistently.
  c. Test caregiver instructions.
    i. Assess how clear the Still-Face Procedure guidelines are, based on pilot participants’ opinions.
    ii. Observe whether caregiver can maintain a neutral expression during the Still-Face episode and follow instructions (e.g., minimal facial touching).
  d. Test additional experiment features (if used).
    i. Check the overall duration of each SFP phase (Play, Still-Face, Reunion) to ensure it is comfortable for participants and yields sufficient data.
    ii. Observe how well infants tolerate the procedure and whether distress levels remain manageable.
    iii. If you plan to incorporate extra tasks (e.g., a baseline or post-SFP caregiver–infant free play), evaluate whether the additional tasks fit smoothly into the session.
  e. Account for potential confounding factors.
    i. Monitor environmental issues (temperature shifts, reflections) that interfere with thermal readings.
    ii. Note motion artefacts, blurred frames, and out-of-focus segments in thermal data.
    iii. Track scheduling constraints (e.g., naps, feeding) that affect infant mood.
14. Conduct the pilot experiment on a small sample size.
  a. Recruit 2–3 dyads initially to evaluate the core feasibility of the procedure (camera setup, time needed per session).
  b. Include additional dyads (up to 5–10) if you wish to inspect preliminary physiological or behavioral outcomes.
  c. Run through the full SFP protocol (3 phases: Play, Still-Face, Reunion), ensuring participants are informed that they can stop if their infant appears overly distressed.
15. Ensure infant well-being:
  a. Ensure the study paradigm protocol is designed to minimize potential distress in infants:
    i. Limit the session duration to 6–10/15 min.
    ii. Allow breaks if needed.
  b. Adapt the study paradigm based on the infant’s age.
    i. Select the age-appropriate seat for infants (high chair vs. baby bouncer).
  c. Avoid intrusive or invasive procedures that may cause discomfort.
16. Ensure caregiver comfort:
  a. Ensure caregiver is seated comfortably.
  b. Ensure caregiver is informed about the protocol’s structure before the experiment.
  c. Allow caregiver to ask any questions to reduce any anticipatory stress.

***Note:*** Once you review the pilot results, if you encounter equipment issues (e.g., unclear thermal footage, strong reflections), instruction concerns (e.g., caregiver struggling to maintain a neutral expression), excessive infant distress, data quality problems, or other procedural challenges, consult the Troubleshooting section for guidance on adjusting camera positioning, SFP duration, lighting, and environmental factors. You may also find it necessary to simplify instructions or offer clearer verbal/visual cues, shorten or extend the Play, Still-Face, or Reunion phases, and/or vary ambient conditions (18–24°C, noise) to ensure minimal artifacts and strong engagement from both caregiver and infant. If the SFP fails to evoke clear thermal or affective responses, consider comparing alternative setups or procedures to find the most effective approach.

### Participant recruitment

**Timing: 1–3 months (for steps 1 to 2)**

This section covers strategies for identifying, screening, and enrolling eligible caregiver-infant dyads, while ensuring ethical standards and participant safety.

This section describes important aspects to consider for participant recruitment.

17. Determine in- and exclusion criteria for participation (see experiment design).
  a. Specify infant age range, health status, and any additional factors (e.g., thermoregulatory concerns) that might affect ITI data quality.
  b. Decide which caregiver demographic characteristics (e.g., language proficiency) or relationship factors (e.g., paternal or maternal) are relevant.
18. Decide on the recruitment strategy based on your target population.
  a. In clinical contexts, advertise at relevant clinics or hospitals, share flyers, or collaborate with support groups.
  b. For community samples, use social media, caregiving forums, or word of mouth to find eligible participants.
19. Decide whether to provide compensation.
  a. Determine if you will offer monetary compensation, small gifts, or informational materials on infant bonding.
  b. Clearly communicate the nature and amount of compensation in recruitment materials.
20. Ensure that all participants meet eligibility criteria and that it will be safe for them to participate.
  a. Verify that no participant presents conditions that could jeopardize well-being (e.g., severe infant distress during lab sessions, major health concerns).
21. Instruct participants on pre-session guidelines.
  a. Ask caregiver to avoid makeup, lotions, sunscreen, smoking, vigorous exercise, and caffeinated or sparkling beverages for at least two hours before the session.
  b. Ask caregivers to remove eyeglasses during ITI sessions; use contact lenses if possible.
  c. Ask caregiver to ensure that the infant is neither hungry nor too fatigued; if needed, allow time for feeding or a short break before starting.

***Note:*** Makeup, lotions, sunscreen, smoking, vigorous exercise, and caffeinated drinks can all affect skin temperature readings.

#### Participant and setup preparation

**Timing: 30 min**

This section outlines the steps to prepare both the testing environment and participants immediately before data collection, ensuring readiness and comfort.

22. Still-Face Procedure setup.
  a. Ensure minimal disruptions:
    i. Clearly post a “Do Not Disturb” sign on the room door to prevent inadvertent entry.
    ii. Silence any alarms, phones, or external devices that could disturb the session.
  b. Prepare short instructions for the caregiver:
    i. Provide a simple outline describing the three SFP phases: Play, Still-Face, Reunion.
    ii. Emphasize to the caregiver the importance of keeping a neutral facial expression and refraining from vocal/physical contact during the Still-Face phase (except in emergency).
    iii. Do not allow toys or pacifiers during the protocol.
    iv. Inform caregiver that the protocol allows for episode shortening in case of extreme distress and inform the caregiver they can stop the session at any time (i.e., crying that cannot be soothed).
  c. Plan for infant soothing after the session:
    i. Keep any needed comfort items (pacifier, small toy, etc.) hidden but accessible in case the infant becomes overly distressed and the procedure is interrupted.
    ii. Have a safe surface or area (e.g., floor mat) where the infant can be placed for a break once data collection is complete.
23. Set up the room according to the instructions mentioned in the “Setup definition” section.
24. Pre-experiment checks.
  a. Final verification of the setup:
    i. Double-check room temperature (optimally, between 18°C and 24°C) and confirm that windows or vents remain closed during recordings.
    ii. Confirm that each camera (thermal and/or behavioral) is powered, memory cards have sufficient space, and cables or batteries are secure.
    iii. Test a brief 5–10 s recording to confirm good image quality, stable focus, and correct framing.
25. Prepare participants before asking them to seat.
  a. Allow a 10–15 min acclimation period so both caregiver and infant can adjust to ambient temperature (18°C–24°C).
  b. Remind the caregiver not to touch the infant’s face throughout the procedure (see troubleshooting if caregiver inadvertently does so).
  c. Prepare short instructions for the caregiver:
    i. Provide a simple outline describing the three SFP phases: Play, Still-Face, Reunion.
    ii. Emphasize to the caregiver the importance of keeping a neutral facial expression and refraining from vocal/physical contact during the Still-Face phase (except in emergency).
    iii. Toys or pacifiers are not allowed during the protocol.
    iv. Inform caregiver that the protocol allows for episode shortening in case of extreme distress and inform the caregiver they can stop the session at any time (i.e., crying that cannot be soothed).
  d. Plan for infant soothing after the session:
    i. Keep any needed comfort items (pacifier, small toy, etc.) hidden but accessible in case the infant becomes overly distressed and the procedure is interrupted.
    ii. Have a safe surface or area (e.g., floor mat) where the infant can be placed for a break once data collection is complete.

***Note:*** Touching the infant’s face during the session can alter heat distribution and affect thermal readings.

**CRITICAL:** Always prioritize the infant’s well-being. If distress exceeds typical levels during the Still-Face phase, consider shortening or concluding the procedure.

26. Adjust and calibrate the equipment, according to the instructions indicated in the “Setup definition” section. Ensure that the thermal imager has been properly calibrated by the manufacturer or an accredited laboratory and verify this by checking the calibration documentation or certificates provided with the device.
27. Pre-experiment checks.
  a. Final verification of the setup:
    i. Check again the room temperature and confirm that windows or vents remain closed during recordings.
    ii. Confirm that each camera (thermal and/or behavioral) is powered, memory cards have sufficient space, and cables or batteries are secure.
    iii. Test a brief 5–10 s recording to confirm good image quality, stable focus, and correct framing.

### Data collection

**Timing: 1–2 h per dyad (depending on your specific procedure)**

This section describes the procedures for running the Still-Face Procedure with thermal and video recording, from consent to the end of the session.

28. Obtain informed consent from the adult participant.
  a. Explain the main study objectives as thoroughly as the study design allows.
  b. Explain the risk factors of the experiment.
    i. Mention the non-invasive and contactless nature of ITI.
    ii. Mention that the SFP induces mild, transient distress in infants and is generally well tolerated. If an infant exhibits heightened distress, the episode will be shortened.
    iii. Explain data usage, storage, and anonymization practices.
    iv. Underline the voluntary nature of participation and the right to withdraw at any time.
29. Give adult participants instructions:
  a. Ensure participants understand the nature of the SFP, including its three phases, and the requirement for the caregiver to maintain a neutral expression during the Still-Face phase (if neutrality breaks, follow Troubleshooting problem 4 steps).
  b. Explain that they should avoid touching their infant, especially on the face, as it might distort the ITI data.
  c. Explain that they should minimize movement and maintain a neutral posture to enhance the reliability of the recordings.
30. Start the experiment:
  a. Start the video recording on both cameras:
    i. Verify that both the video and audio signals are being captured properly.
  b. Start the recording on the thermal cameras:
    i. Press the record button on the thermal camera.
    ii. Document the session: take note of relevant details, such as ambient temperature, distance from the subject, and any camera adjustments.
    iii. Save the recording using an anonymized identifier, incorporating the dyad code.
    iv. Inform participants that the experiment is about to start.
31. During the experiment:
  a. Time the experiment to be able to give prompt instructions to participants.
  b. Inform participants when they should switch from one phase of the SFP to the other.
  c. Check participants’ performance:
    i. If participants are not completing the task correctly, notify them and, if needed, restate or clarify the instructions. Be sure to pause the experiment before intervening.
    ii. Write down any issues occurring during the experiment.
32. At the end of the experiment:
  a. Stop the recordings:
    i. Stop the recording on the thermal cameras by pushing the record button.
    ii. Stop the video recording on each camera.
33. After the experiment.
  a. Communicate to the participants that the experiment has ended and that they can stand up from their seats.
    i. Allow the caregiver to have some time to calm their infant down if they became fussy during the experiment.
    ii. If needed, leave the caregiver alone with their infant.
  b. Collect any additional data of interest (e.g., behavioral questionnaires).
  c. Offer a debriefing of the experiment.
    i. Explain the goal of the study and remain available for further questions.
    ii. Address possible issues that occurred during the experimental session.
    iii. Offer a debriefing document with a reference via email address, where participants can contact you for further questions.
  d. Return the experiment room to its initial state:
    i. Charge the cameras.
    ii. Reorganize the chairs.
    iii. Restore the setup according to the current hygiene norms (e.g., air the room, clean the seats used by the participants, collect trash…).
34. Save the data:
  a. Check that the thermal and behavioural data were correctly recorded.
    i. Verify that both the video and audio recordings are clear and that both participants are visible and audible.
  b. Save the questionnaire data with an anonymized acronym.
    i. After backing up the videos, delete the recordings from the SD cards and reinsert the cards into the cameras.
  c. Eject the SD cards from the cameras and store the videos with an anonymized identifier.

#### Data preprocessing

This section explains how to prepare thermal and video data analysis, including alignment, trimming, and exporting files in the correct formats.

**Figure 2:**
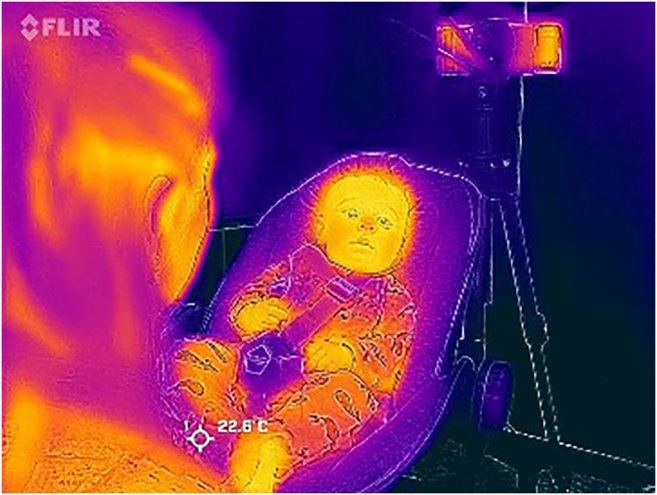
Thermal image of the study toddler during the still-face phase, post-processed to display pixel-specific temperature via colour gradation *Note on ethics*: this is the only participant for whom we have explicit consent to publish an unblurred photograph; we therefore prioritised accurate visual dissemination over facial obscuration, which would have undermined the figure’s purpose. All quantitative analyses were performed on frames that met strict head-movement and image-quality criteria.
  c. Document any preprocessing steps (e.g., time offsets, dropped frames) to maintain data traceability.
    ***Note:*** MP4 video exports from RGB cameras are for behavioral coding; radiometric thermal data should be exported in a format suitable for temperature analysis.

35. Audio-visual data preprocessing:
  a. If you intend to analyze the ITI data alongside the audio-visual data, ensure that both the audio and video begin at the ITI recording start cue.
  b. Align thermal and video data if integrating both for analysis.
    i. Confirm that the start of the thermal recording matches the video timestamp or an explicit synchronization cue (e.g., a clap).
    ii. Trim both recordings so they begin and end at consistent timestamps, if needed.
36. Prepare data for subsequent analyses.
  a. Export the RGB behavioral video in a commonly used format (e.g., .mp4).
    ***Note:*** If the thermal imager also records MP4 preview video, treat this file as distinct from the radiometric data and use it only for visual reference.
  b. Export the thermal data directly from the imager in radiometric format (or as frame-by-frame data via proprietary software) (see Figure 2).
    ***Note:*** In this protocol, thermograms were extracted directly from radiometric still images/video exported from the thermal imager, rather than from RGB MP4 recordings. MP4 video exports from thermal imagers typically do not contain embedded temperature data, and the visual output in MP4 may fluctuate during recording due to automatic NUC and dynamic scale adjustments in response to scene changes. For quantitative analysis, use radiometric image exports or frame-by-frame temperature data.

## Expected outcomes

This protocol is designed to produce synchronised thermal and behavioural datasets that capture fine-grained changes in autonomic and social responses during the Still-Face Procedure (SFP) in caregiver–infant dyads. When executed under the environmental and procedural controls described, researchers can expect to obtain continuous, high-resolution long-wavelength infrared (LWIR) thermal recordings of the caregiver’s and infant’s faces across the Play, Still-Face, and Reunion phases (see Figure 2), accompanied by standard RGB video for behavioural coding. The thermal data should provide stable pixel-level temperature maps, allowing for the extraction of quantitative measures such as mean temperature in specific facial regions of interest (e.g., nasal tip, forehead) and the temporal dynamics of these measures throughout the SFP phases. Behavioural video coding will yield aligned measures of gaze, affect, touch, and other social signals, enabling the investigation of associations between physiological and behavioural changes.

Under optimal conditions, the protocol should yield a high proportion of analysable thermal frames (>80%), minimal motion artefacts, and consistent alignment between thermal and behavioural recordings. Expected patterns may include a decrease in nasal tip temperature during the Still-Face phase, reflecting sympathetic arousal, and a partial temperature rebound during Reunion, accompanied by behavioural signs of re-engagement (e.g., increased gaze to caregiver, positive affect). The resulting datasets will be suitable for within-subject comparisons across phases, between-group contrasts (e.g., clinical vs. non-clinical populations), and integration with other physiological measures. When paired with the preprocessing pipeline described here, these outcomes will allow reproducible extraction of temperature time series, robust statistical modelling, and transparent data sharing in open repositories.

## Quantification and statistical analysis

**Figure 3:**
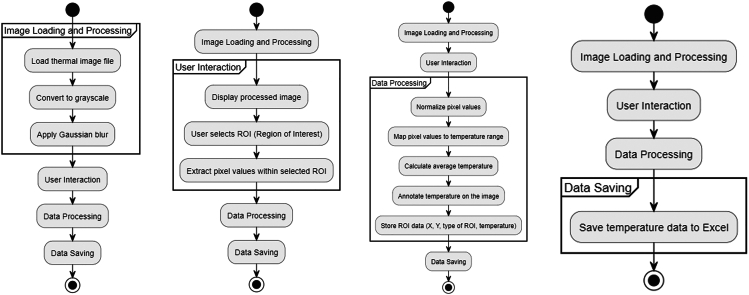
Python pipeline for extraction of temperature data from thermal images

ITI applied to caregiver–infant dyads is a relatively new methodological approach, and there is currently no single standardized analysis protocol for extracting and interpreting thermal data from interactive contexts such as the SFP. Below, we provide an overview of the primary steps you might follow to quantify and statistically analyze thermal signals, drawing on the preprocessing pipeline described above (Figure 3). Researchers are encouraged to adapt these steps based on their specific research questions, available software, and analytic preferences.

Analyses can also be run in FLIR ResearchIR or FLIR Thermal Studio, and free/open-source suites such as Thermimage (R), the ThermoVision plug-in for ImageJ/Fiji, or the InfraRedCam toolbox for MATLAB. For purposes of widespread accessibility, an original application was developed in Python to extract temperature data from thermal images. Here follows a general description of how the application works:

1. Image Loading and Processing:
  a. The thermal image is loaded and converted in grayscale format. In this way, each pixel’s color value ranges from 0 (pure black) to 255 (pure white), which allows for subsequent processing steps. This mapping is only valid if a fixed, known temperature range with a linear look-up table is used in the acquisition. Most thermal imaging systems allow the user to define the acquisition temperature range. For studies involving infants, a temperature range between approximately 17 °C and 42 °C could be recommended, as it adequately covers the expected range of facial skin temperatures.^1^
  b. To account for noise and images of different definition, a Gaussian blur is applied to the image: Each pixel’s value is recalculated as a weighted average of the surrounding pixels, with weights determined by the Gaussian distribution. The extent of the blurring is controlled by the size of the kernel, which defines the number of neighboring pixels included in the calculation.
2. User Interaction:
  a. The image is displayed to the user.
  b. Regions of interest (ROIs) are defined interactively by the user, with predefined shapes and sizes (in our case, we used a rectangular shape for measuring temperature from the forehead, and a circular shape for the nose).
  c. The grayscale intensity values of each pixel within an ROI are normalized (scaled from a range of 0–255 to 0–1) and then mapped to a user-specified temperature range, corresponding to the thermal tool’s measurement capabilities (e.g., 17°C to 42°C in our case). This transformation effectively converts the grayscale image into a numerical temperature matrix, where each pixel’s value represents the actual temperature at that point in the image. This ensures that any subsequent statistical analyses are based on identified temperature data rather than raw pixel intensities.
3. Data Processing:
  a. Subsequently, the average temperature within each ROI is calculated from the numerical temperature matrix within the ROI. .
  b. For visualization purposes, the ROI label and its corresponding temperature value are superimposed on the image.
  c. The data for each ROI, coordinates of the ROI, ROI type, and temperature measured is stored to be subsequently saved in an Excel file.
4. Data Saving:
  a. Finally, when the user closes the image, the data collected from the image is saved in an Excel file.

### Measures

#### Caregiver-infant behaviors

5. Caregiver infant-behaviours can be analysed through the Noldus Observer XT software (see key resources table). Free behavioral-coding alternatives include BORIS, ELAN, OpenSHAPA, and Mangold VideoSync, all of which allow frame-accurate annotation.
6. Exclude frames from quantitative analysis where the infant’s face is significantly obscured (e.g., by hands or extreme head turns), out of focus, or where the camera’s perpendicularity to the face is severely compromised.
7. A trained coder, blind to other study data, should rate the videos according to existing coding system. For Nazzari et al.^1^ study purposes a behavioral coded was adapted from previous studies.^11^^,^^12^ Define a detailed ethogram for each measure with complete operational definitions, evente/duration rules, and inter-rater reliability targets, before coding begins.
  a. The following dimensions were coded:
    i. Infants’ affect: positive, negative and neutral.
    ii. Infants’ Gaze: caregiver-directed, object-directed, hands-directed, or gaze aversion.
    iii. Infants’ Self-comforting behaviours.
    iv. Infants’ Social bids.
    v. Maternal vocalization: no vocalization, negative vocalizations, socio-cognitive vocalizations, nurturing vocalization.
    vi. Maternal touch: no touch, negative touch, scaffolding touch, nurturing touch.
  b. If variables have a low occurrence rate (e.g., <0.5%), they can be excluded from the analysis.

***Note:*** The complete step-by-step behavioral coding protocol is available in the published OSF repository (see key resources table). Reporting the proportion and pattern of excluded frames helps ensure transparency and identify potential biases.

#### Caregiver-infant facial temperature

8. Selection of specific regions of interest (ROIs), based on previous literature.
  a. More frequent ROIs for infants are forehead and nasal tip.^5^^,^^13^
9. Set parameters:
  a. Temperature range set at 17–42°.
  b. Emissivity to 0.98 (human skin).
  c. Sampling rate of 8.7 Hz.
10. Extract frames:
  a. Decide at what frequency to extract frames; every 5s are suggested.^13^
11. Manual selection of ROIs for each frame:
  a. Frames where faces are obstructed, present marked motion artifacts, or result blurred or out of focus, will have to be discarded from analysis.

For the ad-hoc Python script, please refer to the OSF link & Preprocessing data section. Model temporal autocorrelation by either down-sampling to non-overlapping windows that exceed the camera’s frame-to-frame thermal latency (e.g., 1 Hz bins) or fitting mixed-effects models with an error term, ensuring that significance tests are based on independent residuals.

## Limitations

The thermal response to an emotional stimulus is characterized by a relatively delayed onset compared to other physiological measures. Specifically, changes in skin temperature exhibit a latency of approximately 10 seconds, which is considerably slower than skin conductance responses that typically emerge within 3 seconds.^14^^,^^15^ This delayed response may be attributed to the physiological mechanisms governing thermoregulation, which involve a complex interplay of vasomotor activity and metabolic processes. Consequently, researchers utilizing ITI as a measure of emotional or physiological responses should take this inherent latency into account when interpreting temporal dynamics in their data.

Second, if using an entry-level ITI camera, while this device offers portability and affordability, it is important to acknowledge its limitations in terms of precision compared to higher-end ITI systems. In these cases, observed small temperature variations may reflect measurement noise or subtle thermal responses that this methodology, with its inherent limitations, is not optimized to reliably capture.

Third, statistically significant variations in skin temperature might not be metrologically significant. Please refer to new guidelines^16^ to adequately interpret ITI findings.

Fourth, facial muscles movements can influence temperature variations. To overcome this limitation consider simultaneous measurement of facial muscles movements or including control conditions where the infants engaged in motor activities but it is not exposed to the emotional stimuli.

Fifth, the laboratory setting and constraints on maternal behaviour may limit ecological validity. Keep this into account when interpreting the study findings.

Finally, a key methodological consideration in studies utilizing thermal imaging is the sample size. Research in this area often relies on relatively small participant pools, which can limit statistical power and the generalizability of findings. Given the variability in individual thermal responses and the influence of environmental factors such as ambient temperature and humidity, larger sample sizes would be beneficial to ensure robust conclusions. However, the logistical challenges associated with recruiting participants for thermal imaging studies, as well as the need for controlled experimental conditions, may constrain the feasibility of large-scale data collection. Researchers should therefore consider these factors in their study design and, when possible, plan for extended recruitment periods to enhance the reliability of their results.

## Troubleshooting

### Problem 1

Motion artifacts in ITI data: The caregiver or infant moves excessively, causing blurred or distorted thermal images.

### Potential solution

• Instruct the caregiver to maintain a stable posture throughout the procedure, with minimal head movements (see data collection, Step 14).
• If excessive movement occurs, apply advanced motion correction algorithms and/or manual tracking systems in post processing.
• Frames where faces presented motion artifacts or appear blurred need to be discarded from analysis. Keep track of the percentage of rejected frames for each participant.

### Problem 2

Loss of ITI data due to obstruction: The caregiver’s or infant’s face is partially covered, preventing accurate thermal measurements.

### Potential solution

• Ensure hair is tied back and no clothing (e.g., scarves, collars) obstructs the face (see participant recruitment*,* Step 5 and participant and setup preparation, step 9).
• Position the infant in a way that optimizes face visibility (e.g., slightly reclining in a baby bouncer for younger infants).
• Instruct the caregiver to avoid resting their hand on their face or making prolonged contact with the infant’s face, which could alter heat distribution.
• Consider manual tracking of obstructed frames in post-processing. Frames where faces present marked obstructions need to be discarded from analysis. Keep track of the percentage of rejected frames for each participant.

### Problem 3

Inconsistent ITI readings due to room conditions: Sudden changes in ambient temperature or airflow affect the accuracy of thermal recordings.

### Potential solution

• Maintain a constant room temperature between 18°C and 24°C and avoid placing participants near air vents or drafty areas (see setup preparation, Step 7).
• Ensure windows and doors remain closed during the session to prevent temperature fluctuations. Black curtains might be an option to prevent direct sunlight.
• Avoid using additional lighting sources, as they emit infrared radiation that is detected by the thermal imager and included in its measurements. This can significantly distort surface temperature readings, especially when analysing skin temperature, in addition to creating unevenly heated surfaces.
• Allow sufficient warm-up time for the thermal cameras as per the manufacturer’s instructions.

### Problem 4

Caregiver does not fully follow the instructions: The caregiver struggles to maintain a neutral, unresponsive expression and unintentionally interacts with the infant (e.g., smiling, nodding, making eye contact).

### Potential solution

• Provide instructions during the first telephone contact and include them in the informed consent (see data collection, Step 12).
• Before the session begins, review the instructions with the caregiver.
• Remind the caregiver that even minimal reactions (eye contact, small smiles) can alter infant responses and should be avoided.
• If the caregiver does not understand the signal for transition between one episode to another, this can be repeated.
• If the caregiver breaks the still-face, note the deviation in the session log and consider whether the data for that segment should be excluded.
• Use behavioral recordings to check adherence with the experimental instructions.
• Caregiver behaviors during the procedure should be coded as previously detailed in order to ensure the lack of vocal and tactile behavior during the still phase episode (see measures, Step 7).

## Resource availability

### Lead contact

Further information and requests for resources should be directed to and will be fulfilled by the project’s lead contact, Sarah Nazzari (sarah.nazzari@unipv.it).

### Technical contact

Further information and request regarding protocol, procedure, and troubleshooting should be directed to and will be fulfilled by the technical contact, Dr. Mirko Morgese Zangrandi (morgese.mirko@gmail.com).

### Materials availability

This study did not generate new unique reagents.

### Data and code availability

Data and scripts are available via OSF (https://osf.io/8pfx9/). Any additional information required to implement this protocol is available from the project’s lead contact upon request.

## Acknowledgments

We would like to thank Andrea Sandmann from the Max Planck Institute for Human Cognitive and Brain Sciences for her support with the figures. N.H. was supported by the Brazilian National Council for Scientific and Technological Development (CNPq, 420360/2022-0). S.N. was supported by the European Social Fund REACT-EU, PON Ricerca e Innovazione 2014-2020. L.P. was supported by #NEXTGENERATIONEU (NGEU) and funded by the Ministry of University and Research (MUR), National Recovery and Resilience Plan (NRRP), project MNESYS (PE0000006) – A multiscale integrated approach to the study of the nervous system in health and disease (DN. 1553 11.10.2022). Partial support was provided by the Italian Ministry of Health (Ricerca Corrente 2024). Open Access funding enabled and organized by Projekt DEAL.

## Author contributions

All authors contributed equally to the present version of the manuscript.

## Declaration of interests

The authors declare no competing interests.
